# 二维超高效液相色谱-四极杆/飞行时间质谱法解析替考拉宁杂质

**DOI:** 10.3724/SP.J.1123.2022.03044

**Published:** 2023-02-08

**Authors:** Wujun SHAO, Yan’an CHEN, Honglu YUAN, Meichun JIN, Xuefei ZHOU, Yumei QIN, Heyou YANG, Yanling HE

**Affiliations:** 浙江海正药业股份有限公司，浙江 台州 318000; Zhejiang Hisun Pharmaceutical Co.，Ltd.，Taizhou 318000，China

**Keywords:** 二维超高效液相色谱, 四极杆/飞行时间质谱, 替考拉宁, 杂质, 解析, two-dimensional ultra performance liquid chromatography （2D-UPLC）, quadrupole/time-of-flight mass spectrometry （Q/TOF-MS）, teicoplanin, impurity, analysis

## Abstract

建立了二维超高效液相色谱-四极杆/飞行时间质谱法（2D-UPLC-Q/TOF-MS）对替考拉宁组分分离和杂质结构解析的分析方法，有效地解决了流动相中含不挥发性磷酸盐的色谱系统不适用于液相色谱-质谱快速鉴定替考拉宁杂质的难题。一维超高效液相色谱以Octadecyl silica （ODS） hypersil色谱柱（250 mm×4.6 mm， 5 μm）进行色谱分离，以3.0 g/L磷酸二氢钠溶液（pH 6.0）/乙腈=9/1 （v/v）为流动相A、3.0 g/L磷酸二氢钠溶液（pH 6.0）/乙腈=3/7 （v/v）为流动相B进行梯度洗脱；二维超高效液相色谱以Waters ACQUITY UPLC BEH C_18_色谱柱（50 mm×2.1 mm， 1.7 μm）进行脱盐，以0.01 mol/L甲酸铵（pH 6.0）和乙腈为流动相进行梯度脱盐洗脱。质谱在电喷雾离子源、正离子模式下，采用全信息串联质谱（MS^E^）模式采集质谱数据，锥孔气流速50 L/h，锥孔电压60 V，离子源温度120 ℃，雾化气流速900 L/h，雾化气温度500 ℃，毛细管电压2500 V，碰撞能量20~50 eV。根据杂质精确质量数及其二级质谱信息推导其结构，并对替考拉宁主要成分TA_2-2_的裂解规律进行了推导，发现了2个母核特征离子；对《欧洲药典》10.0收录的10个组分及22个杂质组分进行二级质谱分析，发现了3个新杂质组分。采用该法既可以使用一维超高效液相色谱根据相对保留时间进行组分准确定位，也可以使用二维超高效液相色谱-四极杆/飞行时间质谱二级质谱信息快速、简便、灵敏地对杂质进行结构鉴定，为替考拉宁的质量控制和工艺优化提供了一种新思路。

## Abstract (en)

A two-dimensional ultra performance liquid chromatography-quadrupole/time-of-flight mass spectrometry （2D-UPLC-Q/TOF-MS） method was established for the separation and structural analysis of the components in teicoplanin. This method effectively solved the problems associated with chromatographic systems， such as liquid chromatography-mass spectrometry （LC-MS）， which used a non-volatile phosphate buffer as the mobile phase and were not suitable for the rapid identification of impurities. Moreover， this method circumvented the complexities associated with locating and identifying impurities using the original method by re-establishing a chromatographic system suitable for LC-MS. In this study， for one-dimensional （1D） chromatography， the chromatographic separation was performed on an Octadecyl silica （ODS） hypersil column （250 mm×4.6 mm， 5 μm） with gradient elution using 3.0 g/L sodium dihydrogen phosphate buffer （pH 6.0）/acetonitrile=9/1 （v/v） as mobile phase A and 3.0 g/L sodium dihydrogen phosphate buffer （pH 6.0）/acetonitrile=3/7 （v/v） as mobile phase B. The column temperature was maintained at 30 ℃ and an ultraviolet detector was used at 254 nm for analysis. For 2D chromatography， desalting was performed on a Waters ACQUITY UPLC BEH C_18_ column （50 mm×2.1 mm， 1.7 μm） with gradient elution using ammonium formate buffer （pH 6.0） and acetonitrile as the mobile phases. The column temperature was maintained at 45 ℃. The MS data for the components and impurities were collected by positive ion electrospray ionization （ESI） using the full-information tandem MS mode （MS^E^）. The cone and nebulizer gas flow rates were set at 50 and 900 L/h， respectively. The ion source and nebulizer gas temperatures were set at 120 ℃ and 500 ℃， respectively. The ESI and cone needle voltages were set at 2500 and 60 V， respectively. The collision energy was set at 20-50 eV. The molecular formulas of the components and impurities were determined using their exact masses and isotope distributions， and the structural components and impurities of teicoplanin were deduced from their fragment ions according to the fragmentation pathway of the TA_2-2_ component. Moreover， the 10 components reported in the European Pharmacopoeia 10.0 were analyzed and 22 impurities of teicoplanin were identified by 2D-UPLC-Q/TOF-MS. Three new impurities and two characteristic fragment ions of the teicoplanin parent nucleus were detected， and the fragmentation pathway of TA_2-2_ was deduced. Using this method， 1D-UPLC is applicable for the accurate qualification of components based on relative retention times， and 2D-UPLC-Q/TOF-MS is suitable for the rapid identification of the structure of components based on their fragment ions. The results indicate that 2D-UPLC-Q/TOF-MS may be used to analyze the structure of impurities in teicoplanin based on their exact masses， isotope distributions， and fragment ions. The method is rapid， simple， and sensitive， which provides a novel strategy for the quality control and process optimization of teicoplanin.

替考拉宁是由放线菌发酵产生的一系列结构相似的糖肽类抗生素，主要用于治疗严重的革兰氏阳性菌感染^[1]^。替考拉宁结构由七肽苷元母核、*N*-乙酰葡萄糖、甘露糖、*R*-氨基葡萄糖组成^[2,3]^。《中国药典》2020版及《欧洲药典》10.0收录的替考拉宁活性组分有TA_2-1_、TA_2-2_、TA_2-3_、TA_2-4_、TA_2-5_、TA_2-6_，其中TA_2-2_为主要组分，占各组分相对含量的40%以上，其主成分结构如图1^[4,5]^。

**图1:**
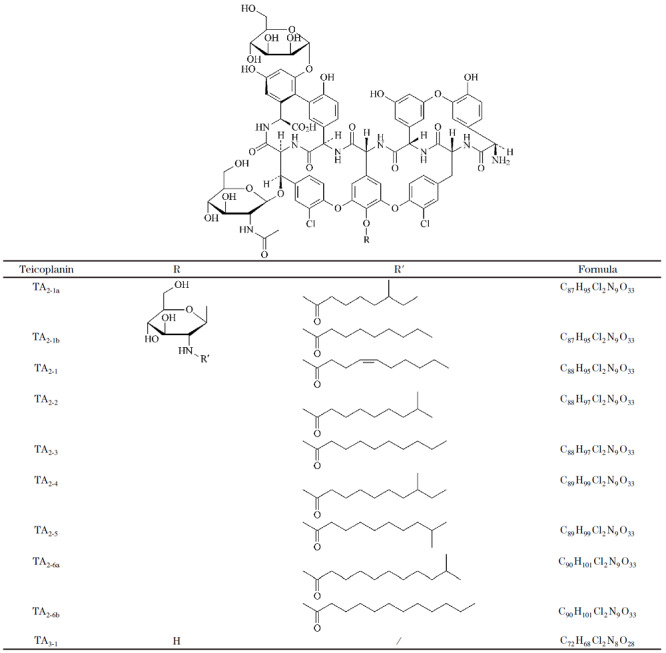
《欧洲药典》中替考拉宁的结构

替考拉宁母核是由7个氨基酸残基之间氧化交联产生的七肽苷元结构，氨基酸上的羟基会进一步糖基化连接*N*-乙酰葡萄糖、甘露糖、葡萄糖胺，葡萄糖胺会继续发生脂肪酰基化，连接的脂肪链的长短、取代基及碳链的不饱和度造成了替考拉宁各组分之间的差异性。目前，对替考拉宁主成分及杂质的研究方法主要有超高效液相色谱-质谱联用、核磁、高效液相色谱-核磁共振联用、气相色谱-质谱联用等技术。Barna等^[6]^通过水解纯化、核磁共振、高分辨质谱等技术对替考拉宁5个组分进行了结构鉴定，为以后替考拉宁其他组分及杂质结构鉴定奠定了基础。Tengattin等^[7]^基于高效液相色谱-质谱方法发现TA_3-1_由TA_2-2_水解脱去*R*-氨基葡萄糖而产生。Cometti等^[8]^通过二维液相色谱-核磁、气相色谱-质谱技术证明TA_2-6a_及TA_2-6b_侧链为十二烷基酰基和10-甲基十一烷基酰基。Borghi等^[9]^通过纯化制备、核磁共振、高分辨质谱等技术证明TA_2-1a_和TA_2-1b_侧链为6-甲基辛酰基和壬酰基。Marrubini等^[10]^建立了液相色谱-质谱方法对替考拉宁主成分及杂质的结构推断，替考拉宁结构由于含有2个Cl，采用低分辨质谱很难显示同位素峰，对某些特征离子碎片的推测也存在偏差。García-Gómez等^[11]^使用不同填料的色谱柱分离替考拉宁组分，通过高分辨质谱推断了14个其他组分的结构。张含智等^[12]^基于高效液相色谱-飞行时间质谱推导了11个具有替考拉宁母核组分的结构和4个*R*-氨基葡萄糖化合物。

近年来，多维或二维液相色谱-质谱联用技术对复杂体系的分离分析是分析化学的重要研究方向。与一维液相色谱-质谱联用相比，多维或二维液相色谱-质谱联用技术既能实现多维或二维液相色谱对复杂体系的分离分析，又能体现质谱高灵敏度及对未知杂质进行定性定量研究的特点，已成为复杂样品分离分析中的一种非常有效的工具。迄今为止，已有很多关于多维或二维液相色谱-质谱联用技术的应用报道，如陈璇等^[13]^使用在线二维集束毛细管柱-高气压光电离-飞行时间质谱测定单萜类化合物；王智聪等^[14]^对金银花中绿原酸和木犀草苷含量的测定；柴爽爽等^[15]^对水稻叶片蛋白质组的研究；徐明明等^[16]^使用在线脱盐质谱法识别了氨基葡萄糖液相色谱的可疑色谱峰等。替考拉宁为发酵药物，成分复杂，杂质较多，《欧洲药典》10.0中替考拉宁分析方法中流动相含有磷酸二氢钠不挥发性盐，与质谱检测的要求不匹配，重新建立适合质谱检测的系统，虽然对检出杂质的结构可以推导，但无法实现与药典色谱条件下各杂质峰的准确定位。因此本实验建立二维超高效液相色谱-四极杆/飞行时间质谱法（2D-UPLC-Q/TOF-MS），对替考拉宁的杂质进行分析，一维液相色谱采用药典方法对各色谱峰进行分离，对《欧洲药典》10.0收录的10个成分根据相对保留时间进行了准确定位，二维液相色谱与质谱联用使用适合质谱检测的流动相体系进行脱盐，对替考拉宁的10个成分进行结构确认，同时对其他未知杂质进行一级、二级质谱分析，确认了张含智等^[12]^推导的11个具有替考拉宁母核组分的结构和4个*R*-氨基葡萄糖化合物，还发现了2个替考拉宁母核特征碎片离子及3个未知杂质，为替考拉宁质量控制和工艺改进提供了参考。

## 1 实验部分

### 1.1 仪器与试药

Waters Acquity UPLC 2D/Xevo G2-XS Q/TOF二维超高效液相色谱-质谱联用仪（美国Waters公司）。Milli-Q Advantage A10超纯水仪（德国Merck公司）。

乙腈为色谱纯（德国Merck公司）；甲酸铵、甲酸均为质谱纯（德国Sigma公司）；其余试剂均为分析纯；欧洲药典委员会替考拉宁对照品（批号：2.0）。

### 1.2 色谱与质谱条件

#### 1.2.1 一维液相色谱方法

色谱柱：Octadecyl silica （ODS） hypersil色谱柱（250 mm×4.6 mm， 5 μm）；波长：254 nm；流速：2.0 mL/min；柱温：30 ℃；进样量：20.0 μL；流动相A： 3.0 g/L磷酸二氢钠溶液（pH 6.0）/乙腈=9/1（v/v），流动相B： 3.0 g/L磷酸二氢钠溶液（pH 6.0）/乙腈=3/7（v/v）。梯度洗脱程序：0~34.50 min， 0B~50%B； 34.50~35.60 min， 50%B~90%B， 35.60~40.25 min， 90%B； 40.25~47.15 min， 90%B~0B； 47.15~51.00 min， 0B。

#### 1.2.2 二维液相色谱方法

色谱柱：Waters ACQUITY UPLC BEH C_18_色谱柱（50 mm×2.1 mm， 1.7 μm）；柱温：45 ℃；流动相A： 0.01 mol/L甲酸铵水溶液（pH 6.0），流动相B：乙腈；流速：0.5 mL/min。梯度洗脱程序：0~2.0 min， 2%B； 2.0~30.0 min， 2%B~80%B； 30.0~35.0 min， 80%B； 35.0~35.1 min， 80%B~2%B； 35.1~38.0 min， 2%B。

#### 1.2.3 质谱条件

电喷雾电离（ESI）源；正离子模式；全扫描范围：*m/z* 100~2500；锥孔气流速：50 L/h；锥孔电压：60 V；离子源温度：120 ℃；雾化气流速：900 L/h；雾化气温度：500 ℃；毛细管电压：2500 V；采集模式：全信息串联质谱（MS^E^）模式；碰撞能量：20~50 eV。

### 1.3 溶液配制

称取替考拉宁20 mg，置于10 mL容量瓶中，用水溶解并稀释至刻度，混匀，配制成质量浓度为2 mg/mL的替考拉宁溶液。

## 2 结果与讨论

### 2.1 一维液相色谱分析

替考拉宁一维液相色谱图中有26个峰（如图2所示），根据《欧洲药典》10.0中的相对保留时间及一级质谱结果，替考拉宁10个主要组分TA_3-1_、TA_2-1a_、TA_2-1b_、TA_2-1_、TA_2-2_、TA_2-3_、TA_2-4_、TA_2-5_、TA_2-6a_、TA_2-6b_的色谱保留时间分别为8.92、17.77、18.42、19.55、20.95、21.50、23.59、24.07、27.12、27.63 min，其他色谱峰均为杂质峰。

**图2:**
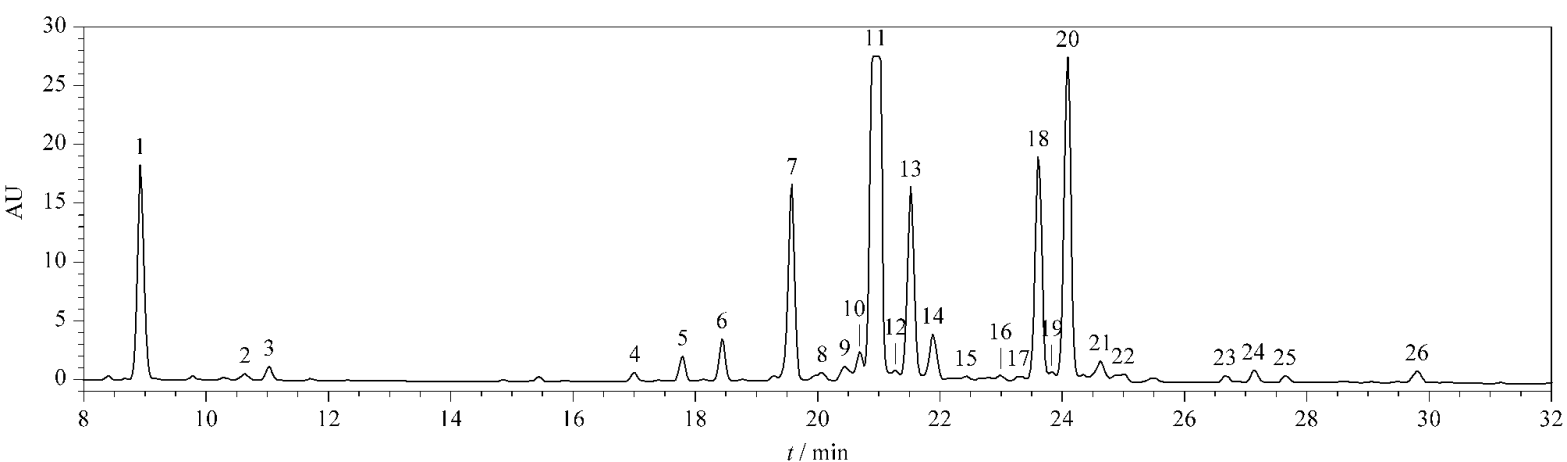
替考拉宁的一维液相色谱图

### 2.2 替考拉宁TA_2-2_裂解途径推导

替考拉宁组分复杂，但其结构相似，在质谱中的裂解途径基本一致，TA_2-2_是替考拉宁含量最多的组分，其结构已知，可以通过其二级质谱信息（如图3所示）来推测替考拉宁其他组分及杂质的裂解方式，TA_2-2_可能的裂解方式见图4。

**图3:**
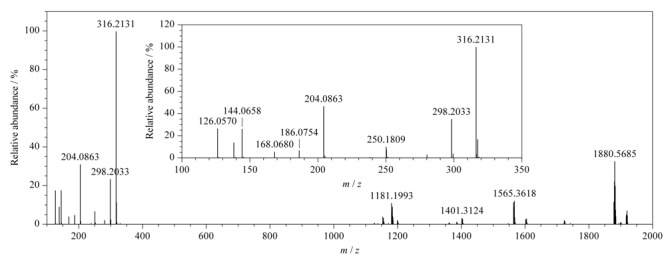
TA_2-2_的二级质谱图

**图4:**
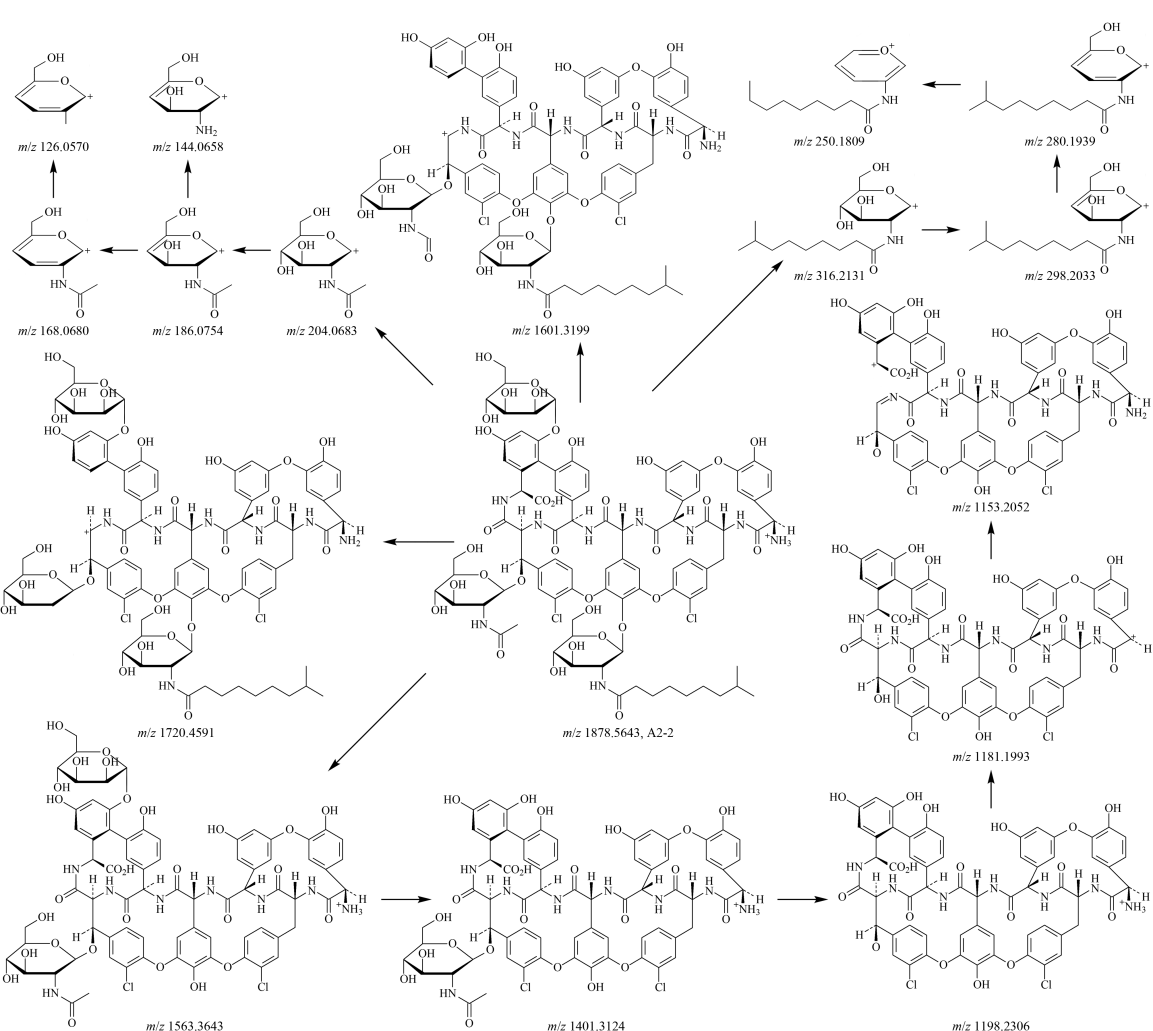
TA_2-2_的质谱裂解规律

TA_2-2_组分（图2中峰11，下文中峰号均与图2中峰号相对应）的准分子离子峰*m/z*为1878.5643（[M+H]^+^）； TA_2-2_组分失去C_5_H_6_N_2_O_4_基团、*R*-氨基葡萄糖、甘露糖、*N*-乙酰葡萄糖后得到*m/z*为1720.4591、1563.3643、1401.3124、1360.2847的替考拉宁母核离子；*m/z* 1401.3124的离子失去氨、*N*-乙酰葡萄糖后得到*m/z*为1384.2740、1198.2306的离子；*m/z* 1198.2306的离子失去氨得到*m/z*为1181.1993的离子。*R*-氨基葡萄糖特征离子（*m/z* 316.2131）失水后得到*m/z* 298.2033的离子，再失去1分子水得到*m/z* 280.1939的离子，该烯醇式离子可以转化成相应的酮式结构*m/z*为250.1809。*N*-乙酰葡萄糖特征离子（*m/z* 204.2863）也会经历相同的失水过程得到*m/z*为186.0754和168.0680的离子。*m/z* 186.0754的离子失去*N*-乙酰基和水得到*m/z* 144.0658的离子，再失去1分子水得到*m/z* 126.0570的离子。

### 2.3 替考拉宁杂质解析

替考拉宁各组分主要区别在于*R*-氨基葡萄糖与糖苷不同，但其裂解规律与主成分TA_2-2_的裂解规律一致，其次结合一维液相色谱的相对保留时间对主成分也可以准确定位。本实验结合相对保留时间及二级质谱信息（主成分及杂质MS数据见表1）确认了《欧洲药典》中的10个组分，并对其他22个杂质进行了裂解分析。

**表1:** 替考拉宁样品中主成分及杂质的MS数据

No.	RRT	[M+H]^+^（*m/z*）	Key fragment ions（*m/z*）	Formula	Name
1	0.43	1563.3555	1462.3145，1401.3124，1384.2893，1360.2847，1198.2306，1181.1993， 1153.2052，204.0863，186.0754，168.0680，144.0658，126.0570	C_72_H_68_Cl_2_N_8_O_28_	TA_3-1_
2	0.51	1563.3555	1462.3145，1401.3124，1384.2893，1360.2847，1198.2306，1181.1993， 1153.2052，204.0863，186.0754，168.0680，144.0658，126.0570	C_72_H_68_Cl_2_N_8_O_28_	TA_3-1_ isomer
3	0.53	1360.2870	1332.2909，1315.2633，1198.2306，1181.1993，1170.2301，1163.1875， 1153.2052，1135.1884	C_64_H_55_Cl_2_N_7_O_23_	TA_3-1_-GlcNAc
4	0.81	1874.5238	1718.4384，1563.3555，1401.3124，1384.2893，1360.2847，1198.2306， 1181.1993，312.1813，294.1691，276.1615，246.1467，204.0863， 186.0754，168.0680，144.0658，126.0570	C_88_H_93_Cl_2_N_9_O_33_	TA_2-1_-2H on R'
5	0.85	1864.5476	1714.4547，1563.3555，1401.3124，1384.2893，1360.2847，1198.2306， 1181.1993，1153.2052，302.2009，284.1866，266.1783，236.1668， 204.0836，186.0754，168.0680，144.0658，126.0570	C_87_H_95_Cl_2_N_9_O_33_	TA_2-1a_
6	0.88	1864.5476	1714.4547，1563.3555，1401.3124，1384.2740，1360.2847，1198.2306， 1181.1993，1153.2052，302.1938，284.1866，266.1783，236.1668， 204.0863，186.0754，168.0680，144.0658，126.0570	C_87_H_95_Cl_2_N_9_O_33_	TA_2-1b_
7	0.93	1876.5498	1720.4591，1563.3551，1401.3087，1384.2853，1360.2803，1198.2231， 1181.2057，1153.2109，314.1924，296.1844，278.1753，248.1634， 204.0889，186.0775，168.0643，144.0668，126.0530	C_88_H_95_Cl_2_N_9_O_33_	TA_2-1_
8	0.96	1673.4567	1656.4471，1153.2191，1360.2847，1198.2306，1181.1993，314.1940， 296.1863，278.1775，248.1661，144.0658，126.0570	C_80_H_82_Cl_2_N_8_O_28_	TA_2-1_-GlcNAc
		1876.5613	1720.4563，1563.3555，1401.3124，1384.2740，1360.2847，1198.2306， 1181.1993，1153.2052，314.1940，296.1863，278.1775，248.1661， 204.0863，186.0754，168.0680，144.0658，126.0570	C_88_H_95_Cl_2_N_9_O_33_	TA_2-1_ isomer
9	0.97	1878.5585	1720.4502，1563.3654，1401.3059，1384.2828，1360.2782，1198.2240， 1181.2068，1153.1986，316.2151，298.1981，280.1888，250.1825， 204.0876，186.0766，168.0638，144.0667，126.0532	C_88_H_97_Cl_2_N_9_O_33_	TA_2-2_ isomer
10	0.99	2040.6152	1959.5892，1883.5231，1802.4960，1725.4077，1563.3669，1546.3328， 1522.3447，1401.3097，1384.2716，1360.2826，1343.2567，1198.2305， 1181.1995，316.2129，298.2032，280.1873，250.1814，204.0873， 186.0766，168.0640，144.0672，126.0540	C_94_H_107_Cl_2_N_9_O_38_	TA_2-2_+mannose
11	1.00	1878.5643	1720.4591，1601.3199，1563.3643，1401.3124，1384.2740，1360.2847， 1198.2306，1181.1993，1153.2052，316.2131，298.2033，280.1871， 250.1809，204.0863，186.0754，168.0680，144.0658，126.0570	C_88_H_97_Cl_2_N_9_O_33_	TA_2-2_
12	1.01	2040.6152	1959.0641，1883.5231，1802.5134，1725.4077，1563.3669，1546.3328， 1522.3287，1401.3097，1384.2716，1360.2977，1343.2567，1198.2305， 1181.2135，316.2129，298.2032，280.1941，250.1814，204.0873， 186.0766，168.0640，144.0672，126.0540	C_94_H_107_Cl_2_N_9_O_38_	TA_2-3_+mannose
		1675.4928	1360.2826，1198.2305，1181.1995，316.2129，298.2032，280.1873， 250.1749，144.0672，126.0540	C_80_H_84_Cl_2_N_8_O_28_	TA_2-3_- GlcNAc
13	1.03	1878.5706	1720.4604，1563.3551，1401.3087，1384.2853，1360.2803，1198.2373， 1181.2057，1153.2109，316.2115，298.2114，280.1917，250.1782， 204.0889，186.0775，168.0643，144.0668，126.0530	C_88_H_97_Cl_2_N_9_O_33_	TA_2-3_
14	1.04	1878.5706	1720.4604，1563.3712，1401.3087，1384.2853，1360.2803，1198.2231， 1181.2057，1153.2109，316.2115，298.2014，280.1917，250.1782， 204.0830，186.0775，168.0643，144.0668，126.0530	C_88_H_97_Cl_2_N_9_O_33_	TA_2-3_ isomer
		1716.5077	1559.4108，1401.3087，1384.2853，1198.2231，1181.2057，1153.2109， 316.2115，298.2014，280.1917，250.1782，204.0830，186.0775， 168.0643，144.0668，126.0530	C_82_H_87_Cl_2_N_9_O_28_	TA_2-2_-mannose
15	1.07	1716.5150	1559.4065，1401.3097，1384.2869，1198.2305，1181.1995，1153.2057， 316.2129，298.2032，280.1873，250.1814，204.0873，186.0766， 168.0640，144.0672，126.0540	C_82_H_87_Cl_2_N_9_O_28_	TA_2-3_-mannose
		1912.5557	1597.3229，1580.2830，1435.2800，1418.2363，1394.2437，1232.1987， 1215.1553，316.2129，298.2032，280.1873，250.1749，207.0873， 186.0766，168.0640，144.0672，126.0540	C_88_H_96_Cl_3_N_9_O_33_	TA_2-2_+Cl
No.	RRT	[M+H]^+^（*m/z*）	Key fragment ions（*m/z*）	Formula	Name	
16	1.10	1844.6136	1529.4078，1512.3693，1367.3529，1326.3231，1164.2623，1147.2397， 1129.2288，316.2129，298.2032，280.1873，250.1814，204.0873， 186.0766，168.0640，144.0672，126.0540	C_88_H_98_ClN_9_O_33_	TA_2-2_-Cl	
		1890.5781	1726.4644，1563.3669，1401.3097，1384.2869，1360.2826，1198.2305， 1181.1995，1153.2057，328.2112，310.2038，292.1938，262.1825， 204.0873，186.0766，168.0640，144.0672，126.0540	C_89_H_97_Cl_2_N_9_O_33_	TA_2-4_ -2H on R'	
17	1.11	1890.5781	1726.4644，1563.3669，1401.3097，1384.2869，1360.2826，1198.2305， 1181.1995，1153.2196，328.2112，310.2038，292.1938，262.1825， 204.0873，186.0766，168.0640，144.0672，126.0540	C_89_H_97_Cl_2_N_9_O_33_	TA_2-5_-2H on R'	
		2054.6470	1890.5248，1809.5022，1725.4247，1563.3669，1546.3328，1522.3447， 1401.3403，1384.2869，1360.2977，1343.2567，1198.2163，1181.1995， 330.2301，312.2173，294.2112，264.1935，204.0873，186.1766， 168.0640，144.0623，126.0540	C_95_H_109_Cl_2_N_9_O_38_	TA_2-4_+mannose	
18	1.13	1892.5868	1728.4777，1563.3551，1546.3364，1401.3087，1384.2853，1360.2803， 1343.2538，1198.2231，1181.2057，1153.2109，330.2292，312.2159， 294.2022，364.1973，204.0889，186.0775，168.0643，144.0668， 126.0530	C_89_H_99_Cl_2_N_9_O_33_	TA_2-4_	
19	1.14	2054.6284	1973.6125，1890.5248，1725.4077，1563.3831，1546.3328，1360.2826， 1343.2417，1181.1995，330.2301，312.2173，294.2042，264.1935， 204.0873，186.0766，168.0640，144.0672，126.0540	C_95_H_109_Cl_2_N_9_O_38_	TA_2-5_+mannose	
20	1.15	1892.5868	1728.4777，1563.3551，1401.3087，1384.2853，1360.2803，1198.2231， 1181.2057，1153.2109，330.2292，312.2159，294.2093，264.1973， 204.0889，186.0775，168.0643，144.0668，126.0530	C_89_H_99_Cl_2_N_9_O_33_	TA_2-5_	
21	1.17	1892.5801	1728.4747，1563.3555，1401.3124，1384.2740，1360.2847，1198.2306， 1181.1993，1153.2052，330.2305，312.2175，294.2042，264.1931， 204.0863，186.0754，168.0680，144.0658，126.0570	C_89_H_99_Cl_2_N_9_O_33_	TA_2-4/5_ isomer	
22	1.19	1730.5262	1566.4255，1401.3097，1384.2869，1198.2305，1181.1995，1153.2057， 330.2301，312.2173，294.2042，264.1935，204.0873，186.0766， 168.0640，144.0672，126.0540	C_83_H_89_Cl_2_N_9_O_28_	TA_2-4/5_-mannose	
23	1.27	1976.6354	1918.5934，1661.4230，1603.3770，1401.3059，1384.2676，1198.2240， 1181.1927，1153.1986，316.2078，298.1981，280.1888，250.1760， 204.0876，186.0766，168.0638，144.0667，126.0532	C_94_H_107_Cl_2_N_9_O_34_	TA_2-2/3_+C_3_H_4_+ C_3_H_6_O on core	
24	1.29	1906.5945	1734.4720，1563.3555，1401.3124，1384.2893，1360.2847，1198.2306， 1181.1993，1153.2052，344.2407，326.2357，308.2357，278.2116， 204.0863，186.0754，168.0680，244.0658，126.0570	C_90_H_101_Cl_2_N_9_O_33_	TA_2-6a_	
25	1.32	1906.5945	1734.4720，1563.3555，1401.3124，1384.2740，1360.2847，1198.2306， 1181.1993，1153.2052，344.2407，326.2357，208.2257，278.2116， 204.0863，186.0754，168.0680，144.0658，126.0570	C_90_H_101_Cl_2_N_9_O_33_	TA_2-6b_	
26	1.42	1918.5992	1760.4945，1756.5317，1603.3997，1441.3494，1400.3171，1181.2134， 316.2131，298.2033，280.1871，250.1809，204.0863，186.0754， 168.0627，144.0658，126.0570	C_91_H_101_Cl_2_N_9_O_33_	TA_2-2/3_+C_3_H_4_ on core	

RRT：ratio of retention time of the impurity to that of TA_2-2_.

峰2的准分子离子*m/z*为1563.3555 （[M+H]^+^），相对保留时间为0.51，推测分子式为C_72_H_68_Cl_2_N_8_O_28_，二级质谱信息与TA_3-1_基本一致，确认为TA_3-1_的同分异构体。

峰3的准分子离子*m/z*为1360.2870 （[M+H]^+^），相对保留时间为0.53，推测分子式为C_64_H_55_Cl_2_N_7_O_23_，二级质谱中未发现*N*-乙酰葡萄糖和*R*-氨基葡萄糖的相关离子，其他质谱信息与TA_3-1_基本一致，确认为TA_3-1_脱去*N*-乙酰葡萄糖的降解产物。

峰4的准分子离子*m/z*为1874.5238 （[M+H]^+^），相对保留时间为0.81，推测分子式为C_88_H_93_Cl_2_N_9_O_33_，其准分子离子*m/z* 1874.5238比TA_2-1_准分子离子*m/z* 1876.5498少2， *m/z*为312.1813、294.1691、276.1615、246.1467的离子均比TA_2-1_ *R*-氨基葡萄糖的相关离子少2； *m/z*为204.0863、186.0754、168.0680、144.0658、126.0570的离子与TA_2-1_中*N*-乙酰葡萄糖基团的相关离子基本一致，推测其R'分子式为C_10_H_15_O，确认为TA_2-1_在R'端脱去2H。

峰8有两个组分，准分子离子*m/z*为1673.4567 （[M+H]^+^）和1876.5613 （[M+H]^+^），相对保留时间为0.96，其中*m/z* 1876.5613的色谱峰二级质谱信息与TA_2-1_基本相同，确认为TA_2-1_同分异构体；准分子离子*m/z*为1673.4567的色谱峰二级质谱中未发现*N*-乙酰葡萄糖基团的相关离子，推测分子式为C_80_H_82_Cl_2_N_8_O_28_，其他二级质谱信息与TA_2-1_基本相同，确认为TA_2-1_脱去*N*-乙酰基葡萄糖的降解产物。

峰9准分子离子*m/z*为1878.5585 （[M+H]^+^），相对保留时间为0.97，其二级质谱信息与TA_2-2_基本一致，根据相对保留时间确认为TA_2-2_同分异构体。

峰10的准分子离子*m/z*为2040.6152 （[M+H]^+^），相对保留时间为0.99，推测分子式C_94_H_107_Cl_2_N_9_O_38_，与TA_2-2_分子式相比多C_6_H_10_O_5_（甘露糖），其中*m/z*为1883.5231、1725.4077、1563.3669、1546.3328、1522.3447的离子与TA_2-2_二级质谱信息*m/z*为1720.4591、1563.3654、1401.3124、1384.2740、1360.2847的离子相比均多162（C_6_H_10_O_5_），其他二级质谱信息与TA_2-2_基本一致，结合相对保留时间确认为TA_2-2_加甘露糖。

峰12有两个组分，准分子离子*m/z*为2040.6152 （[M+H]^+^）和1675.4928 （[M+H]^+^），相对保留时间为1.01，其中*m/z*为2040.6152的色谱峰二级质谱信息与TA_2-2_加一分子甘露糖基本一致，根据相对保留时间确认为TA_2-3_加甘露糖；*m/z*为1675.4928的色谱峰二级质谱中未发现*N*-乙酰葡萄糖基团的相关离子，其他二级质谱信息与TA_2-3_基本相同，结合相对保留时间确认为TA_2-3_脱去*N*-乙酰葡萄糖的降解产物。

峰14有两个组分，准分子离子*m/z*为1878.5706 （[M+H]^+^）和1716.5077 （[M+H]^+^），相对保留时间为1.04， *m/z* 1878.5706的色谱峰二级质谱信息与TA_2-2_基本一致，根据相对保留时间确认为TA_2-3_同分异构体；*m/z* 1716.5077的色谱峰推测分子式为C_82_H_87_Cl_2_N_9_O_28_，与TA_2-2_分子式相比少C_6_H_10_O_5_（甘露糖），其他二级质谱信息与TA_2-2_基本一致，结合相对保留时间确认为TA_2-2_脱去甘露糖的降解产物。

峰15有两个组分，准分子离子*m/z*为1716.5150 （[M+H]^+^）和1912.5557 （[M+H]^+^），相对保留时间为1.07，其中*m/z* 1716.5150的色谱峰二级质谱信息与TA_2-2_脱去甘露糖基本一致，根据相对保留时间确认为TA_2-3_脱去甘露糖的降解产物；*m/z* 1912.5557的色谱峰推测分子式为C_88_H_96_Cl_3_N_9_O_33_，与TA_2-2_分子式相比多1个Cl，准分子离子*m/z*比TA_2-2_多34， *m/z*为1435.2800、1418.2363、1394.2437、1232.1987、1215.1553的离子均比TA_2-2_中相关离子多34，结合相对保留时间确认为TA_2-2_母环上的某个H被Cl取代，但取代位置还有待核磁技术去证明。

峰16有两个组分，准分子离子*m/z*为1844.6136 （[M+H]^+^）和1890.5781 （[M+H]^+^），相对保留时间为1.10，其中*m/z* 1890.5781的色谱峰推测分子式为C_89_H_97_Cl_2_N_9_O_33_，与TA_2-4_分子式相比少2H， *m/z*为328.2112、310.2038、292.1938、262.1825的离子均比TA_2-4_中*R*-氨基葡萄糖相关离子*m/z*少2，其他质谱信息与TA_2-4_基本一致，根据相对保留时间及二级质谱信息确认为TA_2-4_在R'端脱去2H； *m/z* 1844.6136的色谱峰推测分子式为C_88_H_98_ClN_9_O_33_，与TA_2-2_分子式相比少1个Cl， *m/z*为1367.3529、1326.3231、1164.2623、1147.2397、1129.2288的离子均比TA_2-2_中相关离子*m/z*少34，结合相对保留时间确认为TA_2-2_母环上的某个Cl被H取代，但取代位置无法用质谱来证明。

峰17有两个组分，准分子离子*m/z*为1890.5781 （[M+H]^+^）和2054.6470 （[M+H]^+^），相对保留时间为1.11，其中*m/z* 1890.5781的色谱峰二级质谱与峰16（TA_2-4_在R'端脱2H）基本一致，根据相对保留时间确认为TA_2-5_在R'端脱2H； *m/z* 2054.6470的色谱峰推测分子式为C_95_H_109_Cl_2_N_9_O_38_，与TA_2-4_分子式相比多C_6_H_10_O_5_（甘露糖），其中*m/z*为1890.5248、1725.4247、1563.3669、1546.3328、1522.3447的离子与TA_2-4_中相关离子相比*m/z*均多162（C_6_H_10_O_5_），其他二级质谱信息与TA_2-4_基本一致，结合相对保留时间确认为TA_2-4_加甘露糖。

峰19准分子离子*m/z*为2054.6284 （[M+H]^+^），相对保留时间为1.14，二级质谱信息与峰17（TA_2-4_加甘露糖）基本一致，结合相对保留时间确认为TA_2-5_加甘露糖。

峰21准分子离子*m/z*为1892.5801 （[M+H]^+^），相对保留时间为1.17，二级质谱信息与TA_2-5_基本一致，结合相对保留时间确认为TA_2-5_同分异构体。

峰22准分子离子*m/z*为1730.5262 （[M+H]^+^），相对保留时间为1.19，推测分子式为C_83_H_89_Cl_2_N_9_O_28_，与TA_2-5_分子式相比少C_6_H_10_O_5_（甘露糖），其他质谱信息与TA_2-5_基本一致，结合相对保留时间确认为TA_2-5_脱去甘露糖的降解产物。

峰23准分子离子*m/z*为1976.6354 （[M+H]^+^），相对保留时间为1.27，推测分子式为C_94_H_107_Cl_2_N_9_O_34_，与TA_2-2_分子式相比多C_6_H_10_O， *m/z*为126.0532、144.0667、168.0638、186.0766、204.0876的离子与TA_2-2_中*N*-乙酰葡萄糖基团的相关离子基本一致，*m/z*为250.1760、280.1888、298.1981、316.2078的离子与TA_2-2_中*R*-氨基葡萄糖的相关离子基本一致，*m/z* 1918.5934的离子比*m/z* 1976.6354少58（C_3_H_6_O）， *m/z* 1661.4230的离子与TA_2-2_中*m/z* 1563.3643的离子相比多98（C_6_H_10_O）， *m/z* 1603.3770的离子与TA_2-2_中*m/z* 1563.3643的离子相比多40 （C_3_H_4_），结合相对保留时间及二级质谱信息确认为TA_2-2/3_母环上的2个H被丙醇和环丙烷取代，但取代位置还需制备纯化杂质通过核磁精确鉴定。

峰26准分子离子*m/z*为1918.5992 （[M+H]^+^），相对保留时间为1.42，推测分子式为C_91_H_101_Cl_2_N_9_O_33_，与TA_2-2_分子式相比多C_3_H_4_， *m/z*为126.0570、144.0658、168.0627、186.0754、204.0863的离子与TA_2-2_中*N*-乙酰葡萄糖基团的相关离子基本一致，*m/z*为250.1809、280.1871、298.2033、316.2131的离子与TA_2-2_中*R*-氨基葡萄糖的相关离子基本一致，*m/z* 1760.4945的离子比TA_2-2_中*m/z* 1720.4591的离子多40（C_3_H_4_）， *m/z* 1441.3494的离子比TA_2-2_中*m/z* 1401.3124的离子多40（C_3_H_4_），结合相对保留时间及二级质谱信息确认为TA_2-2/3_母环上的1个H被环丙烷取代，但取代位置无法通过质谱确定。

## 3 结论

本研究建立了二维超高效液相色谱-四极杆/飞行时间质谱对替考拉宁杂质的研究方法，对替考拉宁主成分及22个杂质进行了二级质谱分析，方法简便、灵敏，有效地解决了流动相中含不挥发性盐的色谱系统不适用于质谱快速鉴定杂质的难题。本研究新发现了3个杂质TA_2-2_加氯、TA_2-2/3_母环上的2个H被丙醇和环丙烷取代与TA_2-2/3_母环上的1个H被环丙烷取代；*m/z*为1720.4591、1601.3199的2个母核特征离子。研究结果为严格控制替考拉宁质量及替考拉宁工艺纯化提供了一种新解决思路。
